# Changes in Empathy in Patients With Chronic Low Back Pain: A Structural–Functional Magnetic Resonance Imaging Study

**DOI:** 10.3389/fnhum.2020.00326

**Published:** 2020-08-21

**Authors:** Junqin Ma, Xianglong Wang, Qing Qiu, Hongrui Zhan, Wen Wu

**Affiliations:** ^1^Department of Rehabilitation, Zhujiang Hospital, Southern Medical University, Guangzhou, China; ^2^Department of Radiology, Zhujiang Hospital, Southern Medical University, Guangzhou, China; ^3^Department of Physical Medicine and Rehabilitation, The Fifth Affiliated Hospital of Sun Yat-sen University, Zhuhai, China

**Keywords:** empathy, chronic low back pain, functional magnetic resonance imaging, diffusion tensor imaging, brain networks

## Abstract

**Objective**: Many pieces of research have focused on pain within individuals, but little attention has been paid to whether pain can change an individual’s empathic ability and affect social relationships. The purpose of this study is to explore how chronic low back pain changes empathy.

**Methods**: Twenty-four chronic low back pain patients and 22 healthy controls were recruited. We set up an experimental pain-exposed model for each healthy subject. All subjects received a painful-empathic magnetic resonance scan. After the scan, all subjects rated the pain intensity and multiple empathy-related indicators. The clinical assessment scale was the 20-item Basic Empathy Scale in Adults.

**Result**: The chronic low back pain patients reported lower scores on the total scores of BES-A, the subscale scores of emotional disconnection and cognitive empathy, and the discomfort rating. The fMRI results in the chronic low back pain patients showed that there were multiple abnormal brain pathways centered on the anterior insula. The DTI results in the chronic low back pain patients showed that there were reduced fractional anisotropy values in the corpus callosum, bilateral anterior thalamic radiation (ATR), right posterior thalamic radiation (PTR), right superior longitudinal fasciculus (SLF), and left anterior corona radiate (ACR).

**Conclusion**: Our study found that patients with chronic low back pain have impaired empathy ability. The abnormal functional connectivity of multiple brain networks, multiple damaged white matter tracts, and the lower behavioral scores in chronic low back pain patients supported our findings.

## Introduction

Imagine that your hand was accidentally scratched. This experience can lead to nociceptive pain, which originates in the peripheral nociceptors. This stimulus eventually leads to cerebral cortex, causing changes in brain network activity. Now imagine that you saw someone else’s hand accidentally being scratched. This experience typically produces empathy for pain, a phenomenon that, despite differences in origin, has the same characteristics of nociceptive pain and changes in brain network activity.

Briefly speaking, empathy can be generated by directly observing or imagining the emotions of the target. The occurrence of empathy can lead individuals to altruistic and prosocial behaviors towards the plight of others, and this ability to perceive the distress of others also keeps individuals alert to dangerous stimuli (Xiang et al., 2018). Therefore, empathy is of great significance for maintaining social relations and maintaining interactions with others. However, in the eyes of ordinary people, the ability to empathize with others is so common that it seems to be innate in everyone. Many pieces of research have focused on pain, distress, and disability within individuals (Goubert et al., 2005), but little attention has been paid to whether diseases can change an individual’s empathic ability and affect social relationships.

The importance of empathy is also evident in pain medicine. Chronic pain is notorious for harming individuals in many ways. It not only physically tortures patients, but it also stigmatizes them (Cohen et al., 2011). When there is no objective evidence of bodily injury, the subjective pain feeling expressed by patients is often misunderstood as a mental disorder (Cohen et al., 2011). Therefore, the stigmatization has led to the collapse of interpersonal trust between chronic pain patients and others, which is not conducive to social harmony due to the large group of chronic pain patients. Furthermore, on the psychological level, chronic pain can lead to pain catastrophizing and mood disorders such as anxiety and depression. These problems further isolate patients with chronic pain and inhibit the development of prosocial behaviors. Therefore, it is urgent to study the relationship between chronic pain and empathy. A pioneering study by Singer et al. (2004) found that there is a partial overlap between empathy for pain and original pain-activated brain regions. Multiple pain-related brain regions in patients with chronic pain were found to have decreased gray matter volume, altered functional connectivity, and cortical thickness (Hubbard et al., 2014; Seminowicz and Moayedi, 2017). These evidences drive us to link the change of empathy ability to chronic pain.

Previous studies on empathy were mostly behavioral and neuroimaging studies on normal people (Lamm et al., 2011). A few studies have focused on changes in empathy (Roche and Harmon, 2017). Our knowledge is very limited about how empathy has changed in patients with chronic pain. Therefore, the purpose of this study is to explore how chronic pain changes empathy (i.e., what changes have taken place in the empathy ability of patients with chronic pain).

The implicit measures, such as functional magnetic resonance imaging (fMRI), may be a good method to explore empathy. Previous studies have explored the neural brain network mechanism of empathy in healthy adults through fMRI technology and have gotten some excellent results (Engen and Singer, 2013). However, the absence of structural image studies, such as diffusion tensor imaging (DTI), makes the evidence chain of these studies relatively weak, which is one of the limitations of these studies.

Based on the above description, after designating the chronic low back pain (cLBP) group (experimental group) and experimental pain group (EP group), fMRI and DTI technology will be jointly used in this study to explore the brain structural abnormalities and the functional brain changes during empathy state in patients with chronic pain. Diffusion tensor imaging is a magnetic resonance technique that reflects the random diffusion of water molecules in the brain. The Basic Empathy Scale in Adults (BES-A) will be used to quantify the empathy ability of all subjects (Carré et al., 2013). BES-A is usually divided into three subscales, namely, emotional contagion, emotional disconnection, and cognitive empathy. These three components represent three dimensions of empathy. The picture-based paradigm will be used to induce the subjects’ empathy state.

## Experimental Procedures

### Recruitment of Subjects

To reduce confounding factors, we limit the patients included to those with cLBP. We recruited 25 cLBP patients and 25 healthy controls from Zhujiang Hospital of Southern Medical University. For the patients, inclusion criteria were as follows: (1) clinically diagnosed as cLBP (Kreiner et al., 2020); (2) the results assessed by the self-rating anxiety scale (SAS) and self-rating depression scale (SDS) are normal; (3) no fMRI contraindication; (4) did not receive psychological induction training; (5) no cerebral lesions; and (6) no mental or neurological disease. For the controls, inclusion criteria were the same as the patients except for the diagnosis of cLBP, and the demographic characteristics of all subjects (age, gender, and education) were collected.

This experiment was approved by the ethics committee of Zhujiang Hospital of Southern Medical University (Ministry of Health of the PRC, 2018). All subjects signed informed consent. We explained the detailed instructions, experimental procedures, possible risks and discomforts of the study to all volunteers, and answered their questions in detail.

### Procedures

#### Preparation of the Pain Model

To match the patient’s pain status, we set up an experimental pain-exposed model for each healthy subject. The process was as follows: With subjects in a lateral decubitus position, an indwelling needle attached to a 2-ml syringe containing 2 ml of sterile hypertonic (3%) saline was inserted into the lower back muscles, 2 cm to the right of the 4th lumbar vertebra. The time that was taken for the pain caused by the needle puncture to subside ranged from 10 to 30 s in all subjects. After the pain resolved, subjects turned from the lateral decubitus to the supine position, and the hypertonic saline was injected into the lower back muscles of the subjects through the indwelling needle at a speed of 0.15 ml/min, maintaining pain until all scans were completed.

#### Scanning Materials Preparation

The best bottom-up input to produce emotional resonance may be to observe the facial expression of the target directly (Goubert et al., 2005). Besides, many previous studies used photos of limbs with noxious stimuli as empathic stimuli (Gu and Han, 2007; Gu et al., 2010). Consequently, photographs related to pain with both facial expressions and body language may be better experimental stimulus materials for empathy. Before the experiment, we collected photographs that met the above features from various online picture websites. We then put more than 100 questionnaires into society to screen out photos that can cause public empathy. The photos screening paradigm through questionnaire survey was based on a previous pioneering study (Jackson et al., 2005). We finally screened 24 photos as experimental stimulus materials (see Supplementary Figure S1, Table S4 for photo screening). The scanning process was fully explained to each subject and they were instructed to focus on the content of the stimululs materials rather than other things during the scanning process.

#### Scanning Process

Brain scanning consisted of three sessions. First, the T1 sequence was performed. Second, the brain functional sequence was performed and the duration of this session was 6 min. In this session, the stimulus materials were displayed in two consecutive rounds for each participant, and each photo was presented for 7.5 s. Third, each subject underwent a DTI sequence scan lasting 8 min.

#### Postscan Tests

Immediately after the scan, each subject was asked to rate their current pain intensity, discomfort ratings (“How uncomfortable were you when you saw these photos?”), and the intensity of pain suffered by the individual in each photo (“How painful do you think the person in the photo is?”). The assessment of pain intensity of oneself and others can be used to measure the cognitive-evaluative dimension of subjects. The evaluation of the degree of discomfort after receiving stimulation can be used to measure the emotional dimension of empathy (Rutgen et al., 2015). The visual analogue scale (VAS) ranging from 0 to 10 was used to quantify these types of evaluations, for which 0 was painless or no discomfort, and 10 was the maximum intensity of pain or discomfort. The subjects also completed the 20-item BES-A. Each item had a score ranging from 1 to 5, for which one was completely inconsistent, and five was completely consistent. The 20 items were equally divided into three subscales: emotional contagion, emotional disconnection, and cognitive empathy, so that the empathy capacity of subjects could be quantified from different dimensions.

### Data Acquisition and Processing

#### Data Acquisition

All image data were collected by a Philips 3.0 T Achieva magnetic resonance imager in Zhujiang Hospital, Southern Medical University, and scanned in a standard radio-frequency head coil. T1 data were acquired with a T1-weighted rapid spin echo sequence: repetition time (TR)/echo time (TE) = 25/3 ms; flip angle = 90°; field of view (FOV) = 220 mm × 220 mm; matrix size = 256 × 256; 0.859 mm × 0.859 mm in-plane resolution; slice thickness = 5 mm; 24 slices; slice gap = 0.7 mm. Functional MRI data were acquired using a T2*-weighted, single-shot, gradient-recalled echo planar imaging (EPI) sequence, TR/TE = 2,000/35 ms; field of view (FOV) = 230 mm × 230 mm; matrix size = 64 × 64; flip angle = 90°; 3.4 mm × 3.4 mm in-plane resolution; slice thickness = 5 mm; 24 slices; slice gap = 0.7 mm; number of signals averaged (NSA) = 1. DTI data were acquired using a single-shot EPI sequence: TR/TE = 12,500/112 ms; FOV = 256 mm × 256 mm; matrix = 128 × 128; 2 mm × 2 mm in-plane resolution; number of slices = 75; slice thickness of 2 mm and no gap. There were 33 images acquired for each scan: 32 diffusion-weighted images (*b* = 1,000 s/mm^2^) and 1 non-diffusion-weighted image (*b* = 0 s/mm^2^).

#### DTI Data Preprocessing

The Pipeline for Analyzing braiN Diffusion imAges toolbox (PANDA^1^) was used to preprocess the DTI data (Cui et al., 2013). PANDA is a toolbox designed for pipeline processing of diffusion MRI images implemented in MATLAB. Pre-processing included DICOM data conversion, skull removal (the threshold was 0.25), correction of eddy current distortion, and head motion. A voxel-wise tensor matrix map and fractional anisotropy results were obtained for each subject after producing diffusion metrics.

#### Tract-Based Spatial Statistics

Tract-Based Spatial Statistics (TBSS; Smith et al., 2006) were employed to evaluate voxel-based whole-brain white matter measures and the values of fractional anisotropy (FA, one of the important measures of water molecule diffusion, which characterizes the anisotropy of water molecule diffusion and can reflect the integrity of the myelin sheath and axon membrane). The TBSS analysis was carried out using the FMRIB software library (FSL 4.1.4^2^). Briefly, all FA images were nonlinearly registered to the FMRIB58_FA template space. The mean FA image and the white matter tract skeleton (FA threshold was 0.2 to exclude non-WM voxels) were then created. Each subject’s aligned FA image was then projected onto this skeleton. Finally, the Johns Hopkins University ICBM-DTI-81 White-Matter atlas^3^ provided in the FSL toolbox was overlaid on the white matter skeleton, and the FA values of 50 WM regions of interest (ROIs) defined in this standard space were extracted.

#### fMRI Data Preprocessing

The fMRI image data were pre-processed with the Data Processing Assistant for Resting-State fMRI (DPARSF^4^) on the MatlabR2014a platform. Pre-processing included DICOM data conversion, removing the first 10 time points, correcting slice-timing, realignment, nuisance regressors, spatial normalization, smoothing, linear de-trending, and filtering. The first 10 volumes of each scan were discarded to eliminate the instability of the machine magnetic field and the maladjustment of the subject. The motion time courses were used to select subjects’ head movements of <2 mm in translation and 2° in rotation, which were used for further analysis. The nuisances including white matter signals, cerebrospinal fluid signals, and global signal were removed. The images of each subject were registered to the standard plane echo imaging template and resampled at a resolution of 3 mm × 3 mm × 3 mm. The normalized functional images were smoothed spatially using a 6-mm full width at half maximum (FWHM) Gaussian kernel. Finally, linear de-trending was used to reduce the effects of very low-frequency drift and filtering was used to retain the low-frequency band (0.01–0.08 Hz).

#### Location of ROI in Functional Image

As the core brain region of pain empathy, the anterior insula (AI) plays an important role in the emotion-cognitive empathy network. Therefore, locating the ROI in the AI is an important means to explore the joint nodes of the brain network of empathy for pain. In our study, the specific ROI coordinate of AI (*x* = −32, *y* = 25, *z* = 9) was based on our DTI data analysis (for detailed information, see Supplementary Materials), and a spherical ROI with a radius of 3 mm centered on the MNI coordinate was generated.

#### Functional Connectivity Analysis

We used the functional connectivity (FC) function in the rest toolkit^5^ to perform a functional connectivity analysis of the time series of the ROI and the time series of each voxel within the brain. Then, the Pearson correlation coefficients between the time series of brain voxels were obtained, and the Fisher’s *r*-to-*z* transform was used to convert the correlation coefficients into *Z*-scores to obey the normal distribution. ROI-to-whole-brain FC analysis was performed on the patients and healthy controls, based on Gaussian random field theory (GRF) correction (voxel-level *p* < 0.001, cluster-level *p* < 0.05), and the minimum voxel threshold was set to 20. Finally, each subject’s brain *Z*-score image was acquired (Figure 2).

**Figure 1:**
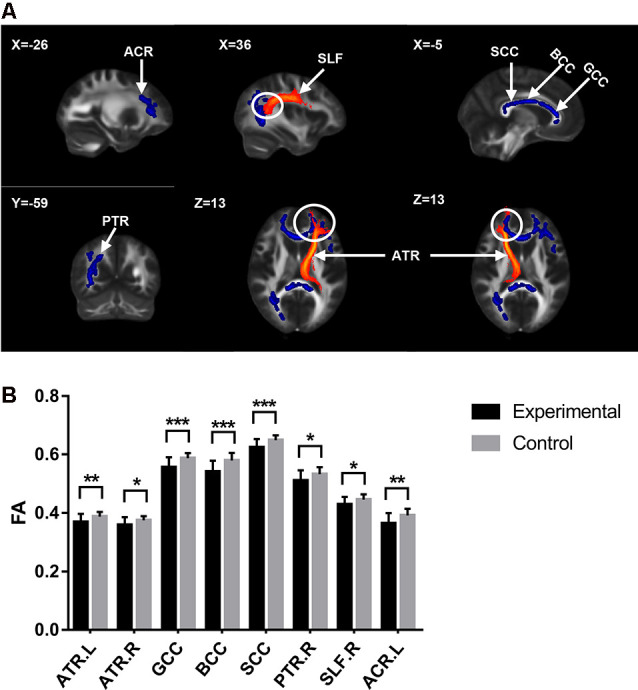
Diffusion tensor imaging (DTI) results with significant difference between groups. **(A)** White matter tracts with significant fractional anisotropy (FA) values change between groups. The upper left number represents the MNI coordinate slice position. Warm tone (red) represents white matter tracts reference system, while cold tone (blue) represents white matter tracts with significant FA values changes between groups. **(B)** The comparison of FA values of white matter tracts with significant differences between groups. Experimental = patients. Control = healty control. *p < 0.05; **p < 0.01; ***p < 0.001.

**Figure 2:**
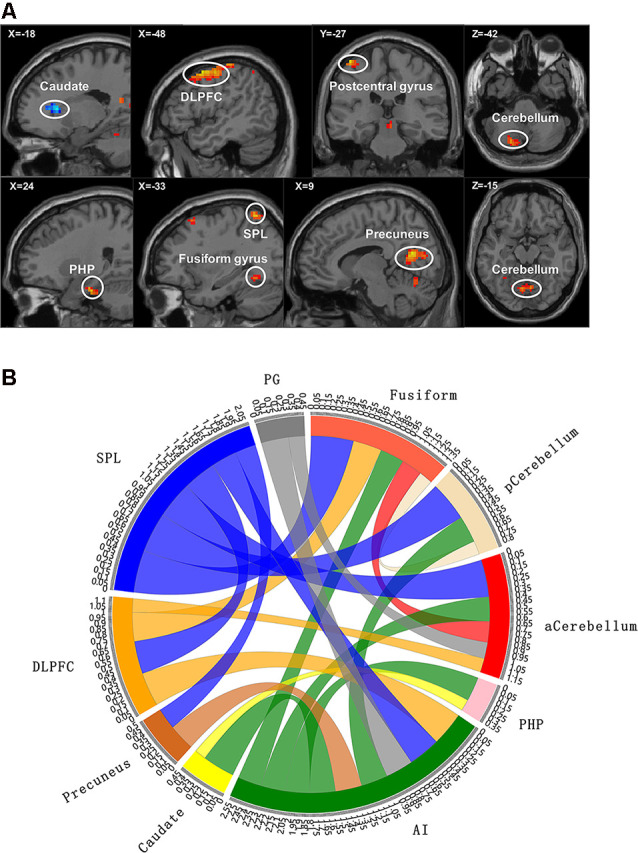
Functional magnetic resonance imaging (fMRI) results with significant difference between groups. **(A)** The brain map of voxel-wise functional connectivity analysis results. The upper left number represents the MNI coordinate slice position. Warm tone (red) represents brain regions with increased functional connectivity strength between groups, while cold tone (blue) represents brain regions with decreased functional connectivity strength between groups. **(B)** Circlize map of functional connectivity between groups from ROI–ROI analysis. The parameters represent the strength of the functional connectivity. PG, postcentral gyrus; aCerebellum, anterior cerebellum; pCerebellum, posterior cerebellum. The parameters represent the functional connectivity (FC) correlation coefficient. Different colors represent different brain regions. For example, yellow represents caudate, and yellow curve represents the FC between caudate and parahippocampal gyrus (PHP).

Based on the results of voxel-wise analysis, each brain region with abnormal functional connectivity to ROI in patients with cLBP was included in the ROI–ROI analysis. These brain regions were set to spherical ROIs with a radius of 3 mm centered on the MNI coordinate point, and the time series of each ROI (including the initial ROI) was extracted and incorporated into the ROI-Wise analysis module in the rest toolkit. We then calculated the correlation coefficient based on time series between the ROIs, and generated an *n* × *n* FC matrix (*n* is the number of ROI). Finally, the statistical analysis of the inter-group FC matrix was carried out.

### Statistical Analysis

The SPSS22.0 (SPSS, Chicago, IL, USA) software package was used to conduct statistical analysis on the demographic characteristics, BES-A scores, and behavior data of subjects in both groups. Two independent sample *t*-tests were used for comparing the data between groups. Non-parametric tests were used for data that were not normally distributed. Pearson correlation analysis was used to calculate the correlation between the FC correlation coefficient with the behavioral results (Spearman correlation analysis for data that did not obey the normal distribution). Chi-square test was used for comparing the dichotomous variables between groups. All statistical assessments were two-tailed, and the significance threshold was *p* < 0.05. The results satisfying normal distribution were expressed as means ± standard deviations; otherwise, they were expressed as median (InterQuartile Range). The effect of age on behavioral data is shown in the Supplementary Tables S1, S2.

The FMRIB software library was used for statistical analysis of DTI data, the significance threshold for intergroup differences was *p* < 0.05 [family-wise error (FWE) corrected for multiple comparisons performed by permutation test with threshold-free cluster enhancement (TFCE)], and the number of permutations was set to 5,000. The rest toolkit was used for statistical analysis of fMRI data, and the significance threshold for intergroup differences was also *p* < 0.05. The resulting images were shown by the rest toolkit, and the FC correlation coefficient between the brain areas was shown by Circlize (Gu et al., 2014). Age was entered into the statistical analysis as a confound regressor in both fMRI data and DTI data.

## Results

### Demographic and Behavioral Data

One patient and three healthy controls were excluded due to poor image quality. There were no significant differences between the groups in education, gender, self-report pain intensity, and others’ pain intensity distribution. The age (*p* < 0.001) and the discomfort rating (*p* = 0.014) distribution between the groups were significantly different (Table 1). For the BES-A scale, there were no significant differences between the groups in the subscale scores of emotional contagion. The total scores (*p* = 0.005), the subscale scores of emotional disconnection (*p* = 0.017), and cognitive empathy (*p* = 0.015) between the groups were significantly different (Table 1).

**Table 1:** Demographic and clinical characteristics and behavioral scores of the participants.

	Patients (*n* = 24)	Controls (*n* = 22)	Test statistics	*P*-value
Demographic features				
Age, years	39.17 ± 8.36	24.91 ± 2.96	7.567	<0.001
Education, years	13.17 ± 1.63	13.23 ± 1.72	−0.123	0.903
Sex ratio, male	9 (37.5%)	8 (36.4%)	0.006*	0.937
Clinical features				
Duration of pain, months				
3–6 months	4 (16.7%)	NA	NA	NA
7–12 months	2 (8.3%)	NA	NA	NA
>12 months	18 (75%)	NA	NA	NA
Pain symptom severity				
Pain intensity, by VAS	5 (2.5)	5 (3)	−1.414**	0.157
Empathy rating				
Discomfort, by VAS	7 (3.5)	5 (2.5)	−2.425**	0.014
Other pain, by VAS	8.5 (1.75)	7.85 (2.58)	−1.078**	0.281
BES-A scores				
Emotional contagion	19.71 ± 4.51	21.55 ± 3.13	−1.592	0.119
Cognitive empathy	30.04 ± 6.58	33.95 ± 3.18	−2.530	0.015
Emotional disconnection	20.79 ± 5.55	23.95 ± 2.65	−2.500	0.017
Total	70.54 ± 13.17	79.45 ± 5.75	−3.017	0.005

**Non-parametric tests. *Chi-square test. The results satisfying normal distribution were expressed as means ± standard deviations; otherwise, they were expressed as median (interquartile range). NA, not applicable; VAS, visual analogue scale (ranging from 0 to 10). Other pain = “How painful do you think the person in the photo is?” Discomfort = “How uncomfortable were you when you saw these photos?”.

### Correlation Analysis

**Figure 3:**
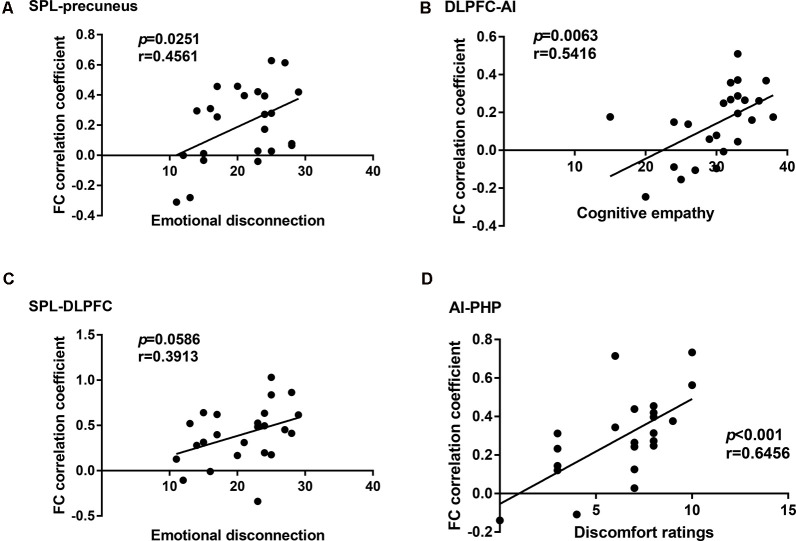
Correlation analysis between the functional connectivity and the behavioral results in the chronic low back pain (cLBP) group. **(A)** Correlation analysis between the subscale scores of emotional disconnection and the functional connectivity coefficient of superior parietal lobule (SPL)-precuneus. **(B)** Correlation analysis between the subscale scores of cognitive empathy and the functional connectivity coefficient of dorsolateral prefrontal cortex-anterior insula (DLPFC-AI). **(C)** Correlation analysis between the subscale scores of emotional disconnection and the functional connectivity coefficient of SPL-DLPFC. **(D)** Correlation analysis between the subscale scores of discomfort ratings and the functional connectivity coefficient of AI-PHP.

In the cLBP group, the functional connectivity between the AI and right parahippocampal gyrus (PHP) showed a significant correlation with the discomfort ratings (*p* < 0.001, *r* = 0.6456), the functional connectivity between the AI and left dorsolateral prefrontal cortex (DLPFC) showed a significant correlation with the cognitive empathy scores (*p* = 0.0063, *r* = 0.5416), the functional connectivity between the left superior parietal lobule (SPL) and right precuneus showed a significant correlation with the emotional disconnection scores (*p* = 0.0251, *r* = 0.4561), but no correlation was found between the SPL-DLPFC functional connectivity and the emotional disconnection scores (Figure 3). No correlation was found between the BES-A scores and the FA values (Supplementary Table S3).

### fMRI and DTI Results

The cLBP group showed significantly reduced FA values in the corpus callosum (CC), bilateral anterior thalamic radiation (ATR), right posterior thalamic radiation (PTR), right superior longitudinal fasciculus (SLF), and left anterior corona radiata (ACR; Table 2 and Figure 1).

**Table 2:** The fractional anisotropy values of the participants.

Regions	Patients	Controls	*t*-value	*P*-value
ATR.L	0.37 ± 0.03	0.39 ± 0.02	−2.897	0.006
ATR.R	0.36 ± 0.03	0.37 ± 0.01	−2.485	0.018
gCC	0.56 ± 0.03	0.59 ± 0.02	−3.921	<0.001
bCC	0.54 ± 0.04	0.58 ± 0.03	−3.977	<0.001
sCC	0.62 ± 0.03	0.65 ± 0.02	−3.621	0.001
PTR.R	0.51 ± 0.04	0.53 ± 0.02	−2.515	0.016
SLF.R	0.43 ± 0.03	0.45 ± 0.02	−2.536	0.015
ACR.L	0.36 ± 0.04	0.39 ± 0.02	−3.040	0.004

*All the data satisfying normal distribution. ATR, anterior thalamic radiation; gCC, genu of corpus callosum; bCC, body of corpus callosum; sCC, splenium of corpus callosum; PTR, posterior thalamic radiation; SLF, superior longitudinal fasciculus; ACR, anterior corona radiata*.

Compared with the EP group, the cLBP group showed increased AI functional connectivity to the left DLPFC, left fusiform gyrus, left SPL, right precuneus, left postcentral gyrus, right PHP, and bilateral cerebellum. The left caudate demonstrated a noticeably decreased connectivity to the AI. The left SPL showed increased functional connectivity to the left fusiform gyrus, left DLPFC, and right precuneus. The left fusiform gyrus showed increased functional connectivity to the left DLPFC. The left caudate showed increased functional connectivity to the right parahippocampal gyrus (Table 3, Figure 2).

**Table 3:** The brain regions with significant functional connectivity strength between groups.

Regions	R/L	BA	Cluster size	MNI			*z* values
			voxels	*x*	*y*	*z*	
Cerebellum posterior lobe	L		42	−21	−75	−42	4.3892
Cerebellum anterior lobe	R		87	6	−69	−15	4.5225
PHP	R	35	21	24	−18	−27	4.7116
Fusiform gyrus	L	37	38	−33	−54	−12	4.1623
Caudate	L	22	37	−18	24	9	−4.2419
Precuneus	R	39	274	9	−63	18	5.1913
DLPFC	L	4/6	100	−48	6	54	5.0656
SPL	L	7	27	−33	−54	63	4.8991
Postcentral gyrus	L	1/2/3	23	−45	−27	66	4.9983

*The minimum voxel threshold was set to 20, based on Gaussian random field theory (GRF) correction (voxel-level *p* < 0.001, cluster-level *p* < 0.05). BA, Brodmann areas; MNI, Montreal Neurological Institute; PHP, parahippocampal gyrus; DLPFC, dorsolateral prefrontal cortex; SPL, superior parietal lobule*.

## Discussion

The empathy for pain has long been a focus of social psychology research. With the progress of research methods and technologies, the study of empathy for pain has developed into a multi-disciplinary and dynamic field, attracting great interest from disciplines such as cognitive psychology, and emotional and cognitive neuroscience. However, as a complex physiological and psychological experience involving multiple dimensions, the exploration of the neurogenesis and development mechanisms of empathy for pain is still in its infancy. At present, there is still a lack of research on whether the progress of some diseases will affect a patients’ ability for pain empathy and then foster social prejudice. In our study, the protocol was specially designed for neuroimaging the change of pain empathy in patients with chronic lower back pain. The behavior measure results showed that there was a significant difference in self-experienced negative emotions (as defined by “discomfort” rating) between the two groups, which is considered to be one of the most specific assessment methods of affective sharing (Singer and Lamm, 2009). For the evaluation results of the BES-A scale, cLBP not only showed a weaker capacity for cognitive empathy but also scored lower on emotional disconnection. The heterogeneity of these behavioral measurement results may provide us with the possibility to combine them with neural measurements for further discussion.

Broadly speaking, empathy is generally composed of cognitive empathy and emotional empathy. Cognitive empathy includes the abilities associated with “mentalizing,” such as “perspective-taking,” “self-other distinction,” and “working memory.” Emotional empathy, on the other hand, involves responding emotionally to the emotional states of others. This so-called “affective sharing,” which involves activation of the “empathy” brain network regarding a certain emotion that underpins the first-hand experience of that emotion (Lamm et al., 2019). As the core brain area of the empathy network, neuroimaging studies have found that the AI plays a key role in the integration of pain, negative emotion processing, affective sharing, sensory coding, and cognitive control (Shackman et al., 2011; Decety et al., 2012). Therefore, AI is activated in both emotional empathy and cognitive empathy, which requires in-depth analysis.

In the field of cognitive empathy, “mentalizing” is an aggregation of various cognitive functions. When cognitive empathy is activated, the activity of several brain areas related to cognitive function enables the brain to analyze the incoming empathy information based on prior knowledge and previous experience, infer the other’s intentions and thoughts, and read the mental state of others without confusing the external information with the own experience, thus producing the top-down empathy regulation effect (Goubert et al., 2005). In our study, cLBP had lower scores of cognitive empathy subscale in BES-A, indicating that cLBP cognitive empathy ability was impaired. However, the term “cognition” is too broad, so it needs to be refined at the neuroscience level. Our results showed that the cLBP’s FC correlation coefficient between the caudate and AI decreased, while that of the AI and DLPFC increased. The cLBP’s FC correlation coefficient between the AI and DLPFC showed a significant correlation with the cognitive empathy scores. Research has found that the caudate is connected to the prefrontal lobe through multiple parallel circuits. One of them, the dorsolateral loop, which connects the DLPFC and caudate, is considered to be closely related to executive function (Kemp et al., 2013). As a high-level cognitive ability, executive function is regarded as the control mechanism of the brain and covers the process of planning, decision-making, judgment, self-regulation, and inhibition. It is essential for goal-oriented behavior and for responding to new events (Chung et al., 2013). Therefore, in this study, we believe that the impairment of cognitive empathy ability in cLBP is related to the low connectivity of the executive function loop. Furthermore, the above discussion seems to be supported by the structural MRI results. The DTI results showed decreased FA values of ACR and genu of corpus callosum (gCC) in cLBP. The damages of ACR and gCC have also been reported in various pain diseases studies (DeSouza et al., 2014; Chong and Schwedt, 2015). As white matter tracts radiate to a wide area of the prefrontal lobe, the decrease of FA values of the ACR and gCC is related to abnormal myelination, axon loss, and inflammation. This means that the functional connection and information transmission between the prefrontal lobe and other brain areas are damaged, which affects the top-down regulation of empathy.

The current study also found that the cLBP had lower scores of emotional disconnection subscale in BES-A. Emotional disconnection is considered to be self-protection regulation mechanism. When witnessing others being hurt, emotional disconnection enables individuals to correctly distinguish the boundaries between themselves and others and to protect individuals against injury and emotional impact (Carré et al., 2013). However, excessive self-protection and resistance to the influence of the external environment will breed indifference. Individuals can easily immerse themselves in their own world and gradually lose the ability to turn their attention outward, which eventually breeds emotional diseases (Hugdahl et al., 2015). In this study, our results showed a circuit with decreased functional connection, which is associated with emotional disconnection, namely, SPL–precuneus–AI–caudate. The cLBP’s FC correlation coefficient between the SPL and precuneus showed a significant correlation with the emotional disconnection scores. The main function of the precuneus is related to “perspective-taking” (Lamm et al., 2011), which allows subjects to generate empathy not only through direct observation but also through imaging other people’s emotions. Furthermore, the precuneus is also involved in the identification and processing of emotional valences in others (Pires et al., 2018). Therefore, we can make an inference in the current study: when patients with cLBP are watching photos, the precuneus with abnormal activities transfers the wrong evaluation information to the executive network, thus affecting the ability to empathize. In addition, the SPL and DLPFC are considered part of the attention network (Corbetta and Shulman, 2002; Fritz et al., 2016). The abnormal functional connection state of SPL and DLPFC caused incorrect attention resources allocation. Although no correlation was found between the SPL-DLPFC functional connectivity and the emotional disconnection scores, there was still a tendency towards statistical significance (*r* = 0.39, *p* = 0.0586). As a result, we can still assume that the cLBP reduced the resource distribution of external attention and allocated more attention resources to self-regulation, which is one of the reasons for the decreased scores of emotional disconnection in cLBP. For structural MRI analysis, the DTI results showed a decreased FA value of the right SLF in cLBP. Research has found that the white matter tract of SLF underlying the temporoparietal cortex and the right temporoparietal junction are considered to be a component of the ventral attention network, a right-lateralized supervisory system (Kucyi et al., 2012). Consequently, the damage of the SLF tracts may affect the distribution of attention resources and then affect the discrimination of self and other emotional states.

A study has proposed that the discomfort ratings of watching photos may be the most appropriate index to evaluate the ability of affective sharing (Singer and Lamm, 2009). In our study, higher discomfort ratings of cLBP represent an abnormal increase in cLBP’s ability to share others’ emotions. The cLBP’s FC correlation coefficient between the AI and PHP showed a significant correlation with the discomfort ratings. The fMRI results showed that the FC correlation coefficient between the fusiform gyrus and AI and that between the AI and PHP were both increased in cLBP. The fusiform gyrus is considered to be an important brain area for facial recognition, and damage to the fusiform gyrus usually results in long-term facial recognition problems (Ghuman et al., 2014). Thus, the fusiform gyrus is the primary activation area of the whole empathy process. The increased functional connectivity between the fusiform gyrus and other brain regions indicated that cLBP enhances the recognition of the face, i.e., increases the bottom-up input. Research has found that the main function of PHP is to extract emotional memory (de Greck et al., 2013). Other studies have found that an individual’s prior experience of pain makes them more likely to develop an empathic response (Goubert et al., 2005), which we consider may be related to deep pain memories. Therefore, in this experiment, we infer that the increase of bottom-up input and the enhancement of pain emotional memory extraction made cLBP more willing to report unpleasant experiences. For structural MRI analysis, the DTI results showed a decreased FA value of ATR and splenium of corpus callosum (sCC) in cLBP. Some studies have found that the ATR is related to the memory and emotional response of autonomic arousal (Huang et al., 2016), while the sCC is closely related to the processing of somatosensory and external input information (Lieberman et al., 2014). The decrease of FA values of these two white matter tracts seems to contradict the fMRI results.

However, a large number of studies have reported that the FA value of the white matter tracts related to somatosensory in patients with chronic pain is decreased (DeSouza et al., 2014; Chong and Schwedt, 2015; Leung et al., 2018), but many patients with chronic pain have symptoms such as hyperalgesia and catastrophizing. Thus, it is worth noting that although the abnormality of the white matter tracts represents the abnormality of related functions, it is not necessarily the decrease. In this study, the damage of the ATR and SCC tracts seems to be consistent with the abnormally enhanced affective sharing ability of cLBP.

## Limitations

Although the results of this study demonstrate the impaired empathy ability of cLBP patients, we still need to pay attention to some shortcomings in this study. First, the sample size was small. The sample size of each group in this study is about 23 cases, which may decrease generalizability. It is necessary to increase the sample size in future experiments. Second, the covariate effect of the age gap on behavior data did not reach statistical significance. However, the significant difference in age distribution between the groups should be avoided in future studies. Third, voxel-based morphometry is useful for studying the damage of chronic pain to the volume of cerebral gray matter, and it could be employed in future experiments. Fourth, selecting multiple ROIs in the same brain area can detect the changes of functional connectivity more thoroughly. Therefore, multiple ROIs and even symmetrical ROIs in brain regions of interest need to be considered in future studies.

## Conclusion

In conclusion, we found that patients with cLBP have impaired empathy ability, which involves cognitive empathy and emotional empathy. We found that the impairment of cognitive empathy is mainly related to the impairment of attention network in patients with cLBP, while the damage of emotional empathy is related to the pain emotional memory. The lower behavioral scores, the abnormal functional connectivity of multiple brain networks, the correlation between behavioral scores and functional connectivity of brain regions, and multiple damaged white matter tracts in chronic pain patients supported our findings. These findings enrich the neural theory of the change of empathy in patients with cLBP. Moreover, we hope that these findings will call attention to the impairment of prosocial behavior in patients with chronic pain.

## Data Availability Statement

The raw data supporting the conclusions of this article will be made available by the authors, without undue reservation.

## Ethics Statement

The studies involving human participants were reviewed and approved by the ethics committee of Zhujiang Hospital of Southern Medical University. The patients/participants provided their written informed consent to participate in this study.

## Author Contributions

All authors had full access to all the data in the study and take responsibility for the integrity of the data and the accuracy of the data analysis. JM and WW: conceptualization, methodology and writing—review and editing. JM and XW: investigation. JM: formal analysis, data curation and writing—original draft. JM, XW, QQ, and HZ: resources. WW: supervision and funding acquisition.

## Conflict of Interest

The authors declare that the research was conducted in the absence of any commercial or financial relationships that could be construed as a potential conflict of interest.

## Footnotes

^1^http://www.nitrc.org/projects/panda

^2^http://www.fmrib.ox.ac.uk/fsl

^3^http://cmrm.med.jhmi.edu/

^4^http://rfmri.org/DPARSF

^5^http://www.restfmri.net/forum/rest

## Abbreviations

fMRI: functional magnetic resonance imaging
DTI: diffusion tensor imaging
FA: fractional anisotropy
BES-A: Basic Empathy Scale in Adults
cLBP: chronic low back pain
VAS: visual analogue scale
TBSS: Tract-Based Spatial Statistics
AI: anterior insula
FC: functional connectivity
CC: corpus callosum
ATR: anterior thalamic radiation
PTR: posterior thalamic radiation
SLF: superior longitudinal fasciculus
ACR: anterior corona radiata
DLPFC: dorsolateral prefrontal cortex
SPL: superior parietal lobule
PHP: parahippocampal gyrus
gCC: genu of corpus callosum
sCC: splenium of corpus callosum.

## References

[B1] Carré A. Stefaniak N. D’Ambrosio F. Bensalah L. Besche-Richard C. (2013). The Basic Empathy Scale in adults (BES-A): factor structure of a revised form. Psychol. Assess. 25, 679–691. 10.1037/a0032297 23815121

[B3] Chong C. Schwedt T. (2015). Migraine affects white-matter tract integrity: a diffusion-tensor imaging study. Cephalalgia 35, 1162–1171. 10.1177/0333102415573513 25712998

[B4] Chung C. Pollock A. Campbell T. Durward B. Hagen S. (2013). Cognitive rehabilitation for executive dysfunction in adults with stroke or other adult non-progressive acquired brain damage. Cochrane Database Syst. Rev. 4:CD008391. 10.1002/14651858.cd008391.pub2 23633354 PMC6464714

[B5] Cohen M. Quintner J. Buchanan D. Nielsen M. Guy L. (2011). Stigmatization of patients with chronic pain: the extinction of empathy. Pain Med. 12, 1637–1643. 10.1111/j.1526-4637.2011.01264.x 22054062

[B6] Corbetta M. Shulman G. (2002). Control of goal-directed and stimulus-driven attention in the brain. Nat. Rev. Neurosci. 3, 201–215. 10.1038/nrn755 11994752

[B7] Cui Z. Zhong S. Xu P. He Y. Gong G. (2013). PANDA: a pipeline toolbox for analyzing brain diffusion images. Front. Hum. Neurosci. 7:42. 10.3389/fnhum.2013.00042 23439846 PMC3578208

[B8] de Greck M. Bolter A. F. Lehmann L. Ulrich C. Stockum E. Enzi B.. (2013). Changes in brain activity of somatoform disorder patients during emotional empathy after multimodal psychodynamic psychotherapy. Front. Hum. Neurosci. 7:410. 10.3389/fnhum.2013.00410 23966922 PMC3744921

[B9] Decety J. Norman G. Berntson G. Cacioppo J. (2012). A neurobehavioral evolutionary perspective on the mechanisms underlying empathy. Prog. Neurobiol. 98, 38–48. 10.1016/j.pneurobio.2012.05.001 22580447

[B10] DeSouza D. Hodaie M. Davis K. (2014). Abnormal trigeminal nerve microstructure and brain white matter in idiopathic trigeminal neuralgia. Pain 155, 37–44. 10.1016/j.pain.2013.08.029 23999058

[B11] Engen H. G. Singer T. (2013). Empathy circuits. Curr. Opin. Neurobiol. 23, 275–282. 10.1016/j.conb.2012.11.003 23219409

[B12] Fritz H. McAuley J. Wittfeld K. Hegenscheid K. Schmidt C. Langner S.. (2016). Chronic back pain is associated with decreased prefrontal and anterior insular gray matter: results from a population-based cohort study. J. Pain 17, 111–118. 10.1016/j.jpain.2015.10.003 26476265

[B13] Ghuman A. Brunet N. Li Y. Konecky R. Pyles J. Walls S.. (2014). Dynamic encoding of face information in the human fusiform gyrus. Nat. Commun. 5:5672. 10.1038/ncomms6672 25482825 PMC4339092

[B14] Goubert L. Craig K. D. Vervoort T. Morley S. Sullivan M. J. de C Williams A. C.. (2005). Facing others in pain: the effects of empathy. Pain 118, 285–288. 10.1016/j.pain.2005.10.025 16289804

[B17] Gu Z. Gu L. Eils R. Schlesner M. Brors B. (2014). circlize implements and enhances circular visualization in R. Bioinformatics 30, 2811–2812. 10.1093/bioinformatics/btu393 24930139

[B15] Gu X. Han S. (2007). Attention and reality constraints on the neural processes of empathy for pain. NeuroImage 36, 256–267. 10.1016/j.neuroimage.2007.02.025 17400480

[B16] Gu X. Liu X. Guise K. Naidich T. Hof P. Fan J. (2010). Functional dissociation of the frontoinsular and anterior cingulate cortices in empathy for pain. J. Neurosci. 30, 3739–3744. 10.1523/JNEUROSCI.4844-09.2010 20220007 PMC2845539

[B18] Huang L. Kutch J. Ellingson B. Martucci K. Harris R. Clauw D.. (2016). Brain white matter changes associated with urological chronic pelvic pain syndrome: multisite neuroimaging from a MAPP case-control study. Pain 157, 2782–2791. 10.1097/j.pain.0000000000000703 27842046 PMC5117992

[B19] Hubbard C. S. Khan S. A. Keaser M. L. Mathur V. A. Goyal M. Seminowicz D. A. (2014). Altered brain structure and function correlate with disease severity and pain catastrophizing in migraine patients. eNeuro 1:e20.14. 10.1523/eneuro.0006-14.2014 25893216 PMC4399775

[B20] Hugdahl K. Raichle M. Mitra A. Specht K. (2015). On the existence of a generalized non-specific task-dependent network. Front. Hum. Neurosci. 9:430. 10.3389/fnhum.2015.00430 26300757 PMC4526816

[B21] Jackson P. Meltzoff A. Decety J. (2005). How do we perceive the pain of others? A window into the neural processes involved in empathy. NeuroImage 24, 771–779. 10.1016/j.neuroimage.2004.09.006 15652312

[B22] Kemp J. Berthel M. Dufour A. Després O. Henry A. Namer I.. (2013). Caudate nucleus and social cognition: neuropsychological and SPECT evidence from a patient with focal caudate lesion. Cortex 49, 559–571. 10.1016/j.cortex.2012.01.004 22325164

[B23] Kreiner D. S. Matz P. Bono C. M. Cho C. H. Yahiro A. (2020). Guideline summary review: an evidence-based clinical guideline for the diagnosis and treatment of low back pain. Spine J. 20, 998–1024. 10.1016/j.spinee.2020.04.006 32333996

[B24] Kucyi A. Moayedi M. Weissman-Fogel I. Hodaie M. Davis K. (2012). Hemispheric asymmetry in white matter connectivity of the temporoparietal junction with the insula and prefrontal cortex. PLoS One 7:e35589. 10.1371/journal.pone.0035589 22536413 PMC3334912

[B25] Lamm C. Decety J. Singer T. (2011). Meta-analytic evidence for common and distinct neural networks associated with directly experienced pain and empathy for pain. NeuroImage 54, 2492–2502. 10.1016/j.neuroimage.2010.10.014 20946964

[B26] Lamm C. Rutgen M. Wagner I. C. (2019). Imaging empathy and prosocial emotions. Neurosci. Lett. 693, 49–53. 10.1016/j.neulet.2017.06.054 28668381

[B27] Leung A. Yang E. Lim M. Metzger-Smith V. Theilmann R. Song D.. (2018). Pain-related white matter tract abnormalities in mild traumatic brain injury patients with persistent headache. Mol. Pain 14:1744806918810297. 10.1177/1744806918810297 30324850 PMC6311536

[B28] Lieberman G. Shpaner M. Watts R. Andrews T. Filippi C. Davis M.. (2014). White matter involvement in chronic musculoskeletal pain. J. Pain 15, 1110–1119. 10.1016/j.jpain.2014.08.002 25135468 PMC4254784

[B2] Ministry of Health of the PRC (2018). Detailed rules for the implementation of regulations on the administration of medical institutions. Available online at: http://www.nhc.gov.cn/fzs/s3576/201808/7a922e4803fa452f99d43a25ec0a3d77.shtml

[B29] Pires F. B. C. Lacerda S. S. Balardin J. B. Portes B. Tobo P. R. Barrichello C. R. C.. (2018). Self-compassion is associated with less stress and depression and greater attention and brain response to affective stimuli in women managers. BMC Womens Health 18:195. 10.1186/s12905-018-0685-y 30482193 PMC6258154

[B30] Roche J. Harmon D. (2017). Exploring the facets of empathy and pain in clinical practice: a review. Pain Pract. 17, 1089–1096. 10.1111/papr.12563 28160400

[B31] Rutgen M. Seidel E. M. Silani G. Riecansky I. Hummer A. Windischberger C.. (2015). Placebo analgesia and its opioidergic regulation suggest that empathy for pain is grounded in self pain. Proc. Natl. Acad. Sci. U S A 112, E5638–E5646. 10.1073/pnas.1511269112 26417092 PMC4611649

[B32] Seminowicz D. A. Moayedi M. (2017). The dorsolateral prefrontal cortex in acute and chronic pain. J. Pain 18, 1027–1035. 10.1016/j.jpain.2017.03.008 28400293 PMC5581265

[B33] Shackman A. J. Salomons T. V. Slagter H. A. Fox A. S. Winter J. J. Davidson R. J. (2011). The integration of negative affect, pain and cognitive control in the cingulate cortex. Nat. Rev. Neurosci. 12, 154–167. 10.1038/nrn2994 21331082 PMC3044650

[B34] Singer T. Lamm C. (2009). The social neuroscience of empathy. Ann. N Y Acad. Sci. 1156, 81–96. 10.1111/j.1749-6632.2009.04418.x016 19338504

[B35] Singer T. Seymour B. O’Doherty J. Kaube H. Dolan R. J. Frith C. D. (2004). Empathy for pain involves the affective but not sensory components of pain. Science 303, 1157–1162. 10.1126/science.1093535 14976305

[B36] Smith S. Jenkinson M. Johansen-Berg H. Rueckert D. Nichols T. Mackay C.. (2006). Tract-based spatial statistics: voxelwise analysis of multi-subject diffusion data. NeuroImage 31, 1487–1505. 10.1016/j.neuroimage.2006.02.024 16624579

[B37] Xiang Y. Wang Y. Gao S. Zhang X. Cui R. (2018). Neural mechanisms with respect to different paradigms and relevant regulatory factors in empathy for pain. Front. Neurosci. 12:507. 10.3389/fnins.2018.00507 30087592 PMC6066512

