# Supplementary material for: Changes in Empathy in Patients With Chronic Low Back Pain: A Structural–Functional Magnetic Resonance Imaging Study
Source: Front Hum Neurosci. 2020 Aug 21;14:326. doi: 10.3389/fnhum.2020.00326 (PMC7473423; doi:10.3389/fnhum.2020.00326)
Supplement: Supplementary file 1 [file Data_Sheet_1.docx]

**Supplementary Table 1.** The effect of the age gap between groups on BES - A scores (Covariance Analysis)

| BES - A scores | Mean Square | F value | *P* value |
| --- | --- | --- | --- |
| Emotional disconnection | 65.399 | 3.562 | 0.066 |
| Cognitive empathy | 0.418 | 0.007 | 0.934 |
| Emotional contagion | 29.212 | 1.953 | 0.169 |

| Behaviour scores by VAS | Mean Square | F value | *P* value |
| --- | --- | --- | --- |
| Pain intensity | 3.804 | 1.977 | 0.167 |
| Discomfort | 1.729 | 0.295 | 0.590 |
| Other pain | 2.936 | 1.303 | 0.260 |

**Supplementary Table 2.** The effect of the age gap between groups on behaviour scores that do not satisfying normal distribution (Regression analysis)

**Supplementary Table 3.** The correlation analysis between the BES-A scores and the FA values

|  | Preserved group | Reduced group | Z/t value | *P* value |
| --- | --- | --- | --- | --- |
| White matter FA value | | | | |
| ATR.L | 0.38 (0.04)* | 0.38 (0.02) | -0.221 | 0.825 |
| ATR.R | 0.37 (0.03)* | 0.37 (0.02) | -0.397 | 0.691 |
| gCC | 0.57 (0.05)* | 0.57 (0.04) | -1.104 | 0.270 |
| bCC | 0.56 (0.04)* | 0.55 (0.03) | -1.810 | 0.070 |
| sCC | 0.63 ± 0.03 | 0.62 ± 0.03 | 0.861 | 0.402 |
| ACR.L | 0.37 (0.05)* | 0.37 (0.06) | -0.132 | 0.895 |
| PTR.R | 0.51 ± 0.04 | 0.51 ± 0.03 | -0.014 | 0.989 |
| SLF.R | 0.43 ± 0.03 | 0.43 ± 0.03 | 0.675 | 0.509 |

We defined those with the BES-A score greater than or equal to 75 points (n = 9) as the empathy preserved subgroup and those with the BES-A score less than or equal to 65 points (n = 9) as the empathy reduced subgroup in the cLBP group. * Non-parametric tests.

**Supplementary Table 4.** Results of questionnaire survey on stimulating materials

|  | Figure S1A | Figure S1B | Figure S1C |
| --- | --- | --- | --- |
| Number of evaluators | 184 | 184 | 184 |
| Discomfort ratings | 5.5 (3) | 5 (3) | 7 (3) |
| Other-directed pain ratings | 7 (3) | 7 (3) | 9 (3) |

The results were expressed as median (InterQuartile Range) due to it does not satisfy the normal distribution.

**Supplementary Figure 1.** These images were obtained from several public picture databases that comply with the CC0 protocol. Figure 1A depicts an individual suffering from unbearable neck pain; Figure 1B depicts a serious injury to an athlete's ankle during a match.

**Supplementary Figure 2.** The central location of the damaged ACR white matter tracts.

**The detailed description of the ROI selection.**

Our DTI results found that the cLBP group showed significantly reduced FA values in the left anterior corona radiate (ACR). The ACR is an important white matter tract to ensure the information transmission between prefrontal lobe and insular lobe. The central location of the damaged ACR white matter tracts was located at (x = -32, y = 25, z = 21) (Supplementary Fig.2). In the case of X = -32 and Y =25, the range of transverse-sectional coordinates of the anterior insula is 4 ≤ Z ≤ 14. This identification was based on the Harvard–Oxford subcortical structural atlas as implemented in the FSL toolbox (Desikan et al., 2006). Therefore, we chose the central coordinate (x = -32, y = 25, z = 9), and a spherical ROI with a radius of 3 mm centred on the MNI coordinate was generated.

Desikan, R., Ségonne, F., Fischl, B., Quinn, B., Dickerson, B., Blacker, D., . . . Killiany, R. (2006). An automated labeling system for subdividing the human cerebral cortex on MRI scans into gyral based regions of interest. *Neuroimage, 31*(3), 968-980. doi:10.1016/j.neuroimage.2006.01.021
