# Supplementary figures and images for: Changes in Empathy in Patients With Chronic Low Back Pain: A Structural–Functional Magnetic Resonance Imaging Study
Source: Front Hum Neurosci. 2020 Aug 21;14:326. doi: 10.3389/fnhum.2020.00326 (PMC7473423; doi:10.3389/fnhum.2020.00326)

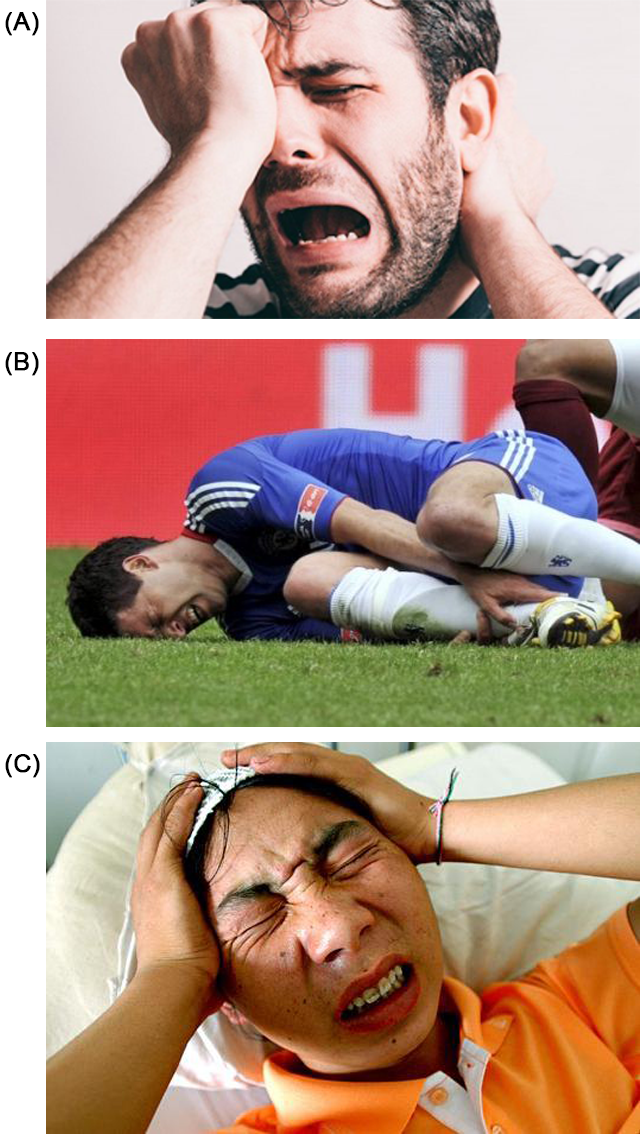

Supplement: Supplementary file 2 [file Image_1.TIF]

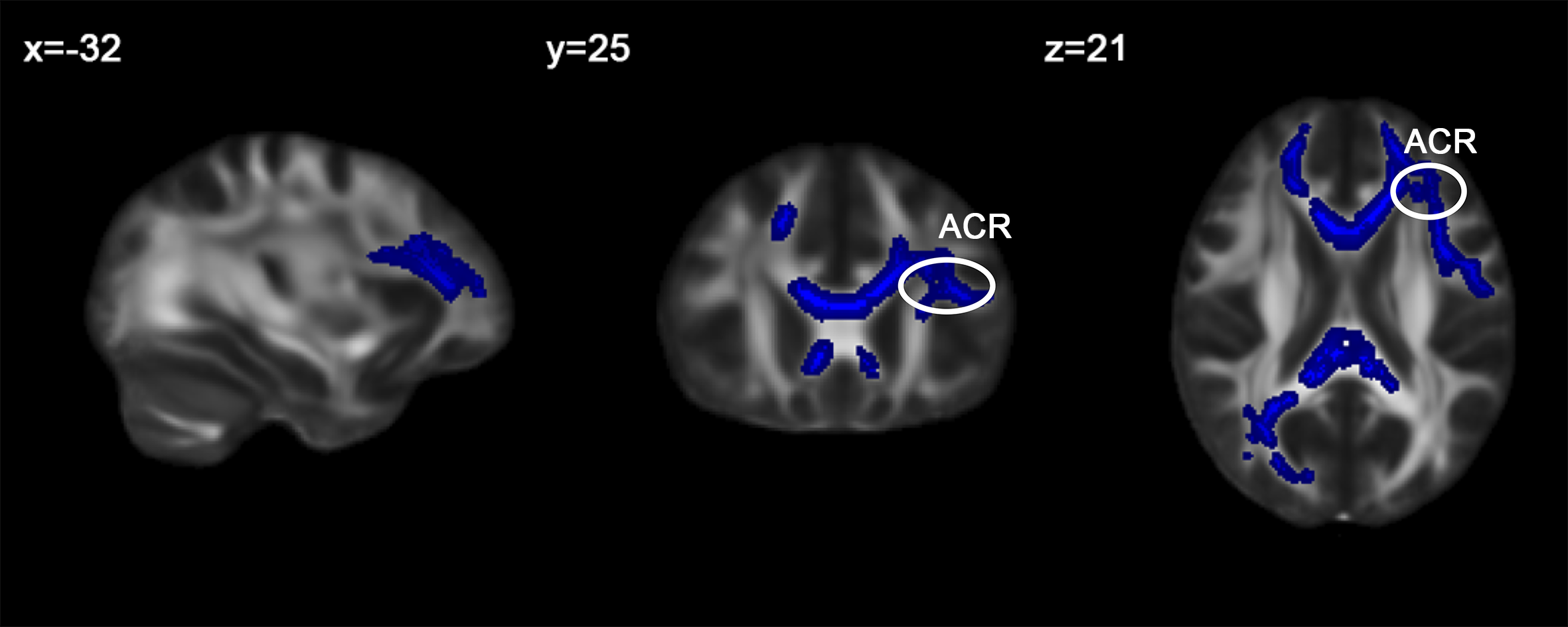

Supplement: Supplementary file 3 [file Image_2.TIF]
